# Inhibition of Retrograde Transport Limits Polyomavirus Infection *In Vivo*

**DOI:** 10.1128/mSphereDirect.00494-17

**Published:** 2017-11-15

**Authors:** Saumya Maru, Ge Jin, Dhimant Desai, Shantu Amin, Shwetank, Matthew D. Lauver, Aron E. Lukacher

**Affiliations:** a Department of Microbiology and Immunology, Penn State College of Medicine, Hershey, Pennsylvania, USA; b Department of Pharmacology, Penn State College of Medicine, Hershey, Pennsylvania, USA; University of Michigan—Ann Arbor; Brown University; University of Michigan Medical School

**Keywords:** T cells, kidney, mouse, mouse polyomavirus, polyomavirus, retrograde transport

## Abstract

Polyomaviruses (PyVs) silently infect most humans, but they can cause life-threatening diseases in immunocompromised individuals. The JC polyomavirus (JCPyV) induces progressive multifocal leukoencephalopathy, a severe demyelinating disease in multiple sclerosis patients receiving immunomodulatory therapy, and BK polyomavirus (BKPyV)-associated nephropathy is a major cause of kidney allograft failure. No effective anti-PyV agents are available. Several compounds have been reported to possess anti-PyV activity *in vitro*, but none have shown efficacy in clinical trials. Productive PyV infection involves usurping the cellular retrograde vesicular transport pathway to enable endocytosed virions to navigate to the endoplasmic reticulum where virion uncoating begins. Compounds inhibiting this pathway have been shown to reduce infection by simian virus 40 (SV40), JCPyV, and BKPyV in tissue culture. In this study, we investigated the potential of Retro-2.1, a retrograde transport inhibitor, to limit infection by mouse polyomavirus (MuPyV) *in vivo*. We found that Retro-2.1 significantly reduced MuPyV levels in the kidney during acute infection without affecting renal function or the MuPyV-specific CD8 T cell response. To approximate the clinical setting of PyV resurgence in immunocompromised hosts, we showed that antibody-mediated depletion of T cells in persistently infected mice elevated MuPyV levels in the kidney and that Retro-2.1 blunted this increase in virus levels. In summary, these data indicate that inhibition of retrograde vesicular transport *in vivo* controls infection in a natural PyV mouse model and supports development of these compounds as potential therapeutic agents for individuals at risk for human PyV-associated diseases.

**IMPORTANCE** PyVs can cause significant morbidity and mortality in immunocompromised individuals. No clinically efficacious anti-PyV therapeutic agents are available. A recently identified inhibitor of retrograde transport, Retro-2^cycl^, blocks movement of PyV virion-containing vesicles from early endosomes to the endoplasmic reticulum, an early step in the PyV life cycle. Retro-2^cycl^ and its derivatives have been shown to inhibit infection by human PyVs in tissue culture. Here, we demonstrate that a derivative of Retro-2^cycl^, Retro-2.1, reduces infection by MuPyV in the kidneys of acutely infected mice. Mimicking the common clinical scenario of PyV resurgence, we further show that MuPyV levels increase in the kidneys of immunocompromised, persistently infected mice and that this increase is inhibited by Retro-2.1. These data provide the first evidence for control of a natural PyV infection *in vivo* by administration of an inhibitor of retrograde transport.

## Abstract (precis)

PyVs can cause significant morbidity and mortality in immunocompromised individuals. No clinically efficacious anti-PyV therapeutic agents are available. A recently identified inhibitor of retrograde transport, Retro-2^cycl^, blocks movement of PyV virion-containing vesicles from early endosomes to the endoplasmic reticulum, an early step in the PyV life cycle. Retro-2^cycl^ and its derivatives have been shown to inhibit infection by human PyVs in tissue culture. Here, we demonstrate that a derivative of Retro-2^cycl^, Retro-2.1, reduces infection by MuPyV in the kidneys of acutely infected mice. Mimicking the common clinical scenario of PyV resurgence, we further show that MuPyV levels increase in the kidneys of immunocompromised, persistently infected mice and that this increase is inhibited by Retro-2.1. These data provide the first evidence for control of a natural PyV infection *in vivo* by administration of an inhibitor of retrograde transport.

## INTRODUCTION

Human polyomaviruses (PyVs) silently infect most humans, but they can cause life-threatening diseases in immunocompromised individuals. PyVs undergo an initial acute infection which is largely controlled by the adaptive immune response, with low levels of virus persisting indefinitely in the kidneys and urogenital tract (1, 2). Uncontrolled replication of the BK polyomavirus (BKPyV) can lead to hemorrhagic cystitis and interstitial nephritis in kidney transplant patients (3–6). BKPyV infection of the urogenital tract can amplify to produce viruria and viremia in kidney allograft patients, resulting in BKPyV-associated nephropathy (BKVAN) in 1 to 10% of cases, with a 30 to 80% incidence of subsequent graft loss (2, 7, 8). No treatment for PyV infections is currently available; although several compounds have shown potent antiviral effects *in vitro*, no compounds have demonstrated therapeutic efficacy in clinical studies (2).

BKVAN is clinically managed by decreasing immune suppression to allow restoration of an immune response to control infection. This treatment modality necessitates tightly balancing viral control and kidney allograft rejection. Potential antiviral agents used to counter PyVs tend to be nephrotoxic (e.g., cidofovir) and often induce acute tubular injury (2). The limited availability of kidneys for transplantation make it a clinical imperative to improve kidney transplant outcomes by minimizing BKVAN-induced graft loss. Patients with lower BKPyV levels can clear the virus more efficiently than those with higher viral loads, suggesting that lowering viral load can enhance host control of infection and prevent subsequent pathology (8).

Multiple steps in the PyV life cycle have been targeted in attempts to develop anti-PyV agents. These anti-PyV agents include agents to impair interaction between virions and host cell surface receptors (9), agents that alkalinize endosomes (10, 11), and agents that interfere with the host cell machinery required for viral genome replication (12–15); however, none of these agents has shown efficacy *in vivo*. The novel compound Retro-2^cycl^ was reported to protect mice from the AB_5_ family of toxins by specifically inhibiting retrograde vesicular transport (16), a cytoplasmic sorting mechanism also used by PyVs to traffic from early endosomes to the endoplasmic reticulum (17). Retro-2^cycl^ has been shown to inhibit BKPyV, JC polyomavirus (JCPyV), and simian virus 40 (SV40) infections *in vitro* with minimal cytotoxicity, and therefore, it has been proposed as a promising candidate compound to control PyV infection (18). Because productive infection by a given PyV is limited to its natural host reservoir, the *in vivo* efficacy of retrograde transport inhibitors has yet to be evaluated in an animal model of PyV infection.

Using an established mouse model of PyV infection, we show that Retro-2.1, a derivative of Retro-2^cycl^ with enhanced potency against AB_5_ toxins (19), inhibited mouse PyV (MuPyV) infection *in vitro* with little impact on host cell viability. In acutely infected mice, Retro-2.1 administered parenterally inhibited MuPyV replication in the kidneys and did so without affecting kidney function or the host anti-MuPyV CD8 T cell response. Antibody-mediated T cell depletion during persistent infection increased viral burden in the kidneys, mimicking natural disease progression in kidney transplant recipients, and this increase was curtailed by Retro-2.1 treatment. Together, these data provide the first evidence that inhibitors of retrograde transport mitigate PyV infection in a natural host.

## RESULTS

### Retro-2.1 inhibits MuPyV infection *in vitro.*

A defining characteristic of polyomaviruses (PyVs) is their tight species specificity, such that productive viral replication is generally limited to the natural host (20–25). Studies of human PyV replication have thus been relegated to tissue culture, with the exception of a JCPyV central nervous system (CNS) infection mouse model in which human glial cells were engrafted into the brains of hypomyelinated Rag^−/−^ mice (26). This inability to evaluate compounds showing anti-PyV activity outside of an *in vitro* setting has stymied efforts to advance anti-PyV agents into the therapeutic realm. We have used MuPyV, a natural mouse pathogen that shares a number of key characteristics with the human PyVs (27), to ask whether inhibition of retrograde transport, which limits human PyV infection *in vitro*, can control PyV infection in mice.

**FIG 1:**
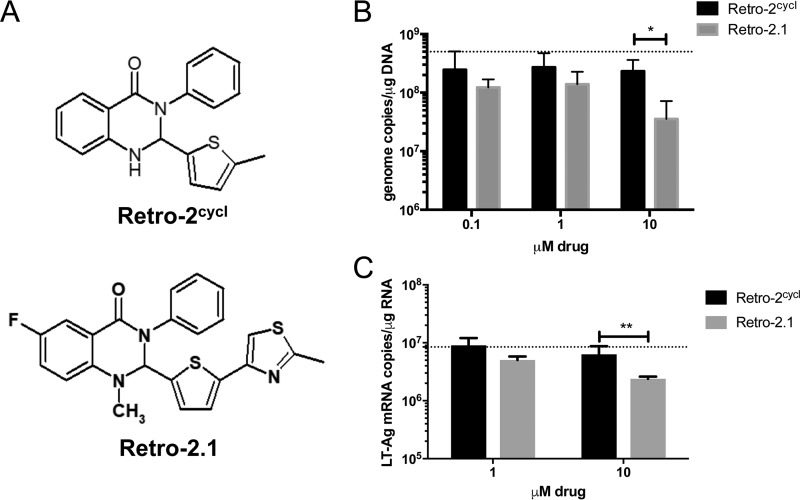
Retro-2.1 inhibits MuPyV replication *in vitro* more effectively than Retro-2^cycl^ does. (A) Chemical structures of Retro-2^cycl^ and Retro-2.1. (B) Subconfluent monolayers of A31 cells were infected at an MOI of 1 for 1.5 h at 37°C. The culture medium was supplemented with the indicated concentrations of Retro-2^cycl^ or Retro-2.1. Total DNA was extracted from cells harvested at 24 h p.i., followed by quantitation of MuPyV genome copies by qPCR. The dotted line depicts the average number of genome copies using a vehicle control. Values are means plus standard deviations (SD) (error bars) for four independent experiments. The values that are significantly different (*P* = 0.0261) are indicated by a bracket and asterisk. (C) Quantitation of LT-Ag mRNA copies at 24 h p.i. under the same infection conditions and treatment with Retro-2^cycl^ or Retro-2.1 as described above for panel B. The dotted line depicts the average number of LT-Ag mRNA copies using a vehicle control. Values are means plus SD for two independent experiments. The values that are significantly different (*P* = 0.0043) are indicated by a bracket and two asterisks.

Retro-2^cycl^, a dihydroxy quinazolinone, was identified as a nontoxic retrograde transport inhibitor with a 50% effective concentration (EC_50_) of 27 µM against Shiga toxin (28), and it has been shown to inhibit replication of human PyVs *in vitro* (18). Derivatives have been synthesized, and structure-activity responses have been evaluated to identify modifications that increase the potency of the parent compound (19, 28, 29). Retro-2.1 contains a 5-ethylthiopene heteroaromatic moiety (Fig. 1A) that lowers the EC_50_ against Shiga toxin to 0.3 µM, which is a 90-fold improvement compared to Retro-2^cycl^ (19). We tested Retro-2^cycl^ and Retro-2.1 for antiviral activity against MuPyV in tissue cultured cells. A31 cells were infected with MuPyV and cultured in the presence of various concentrations of Retro-2^cycl^ and Retro-2.1. At 24 h postinfection (p.i.), MuPyV-infected cells treated with 10 µM Retro-2.1 had significantly fewer viral genome copies (Fig. 1B) than A31 cells treated with Retro-2^cycl^ did. Because viral DNA quantitative PCR (qPCR) assays are unable to distinguish between infectious virions and noninfectious particles, we developed a qPCR assay for large T (LT) antigen mRNA to provide a more accurate measure of the level of infection. As shown in Fig. 1C, Retro-2.1’s antiviral activity for LT transcripts by qPCR mirrors its activity for viral genome copy numbers and strengthens the conclusion that Retro-2.1 inhibits productive viral infection.

**FIG 2:**
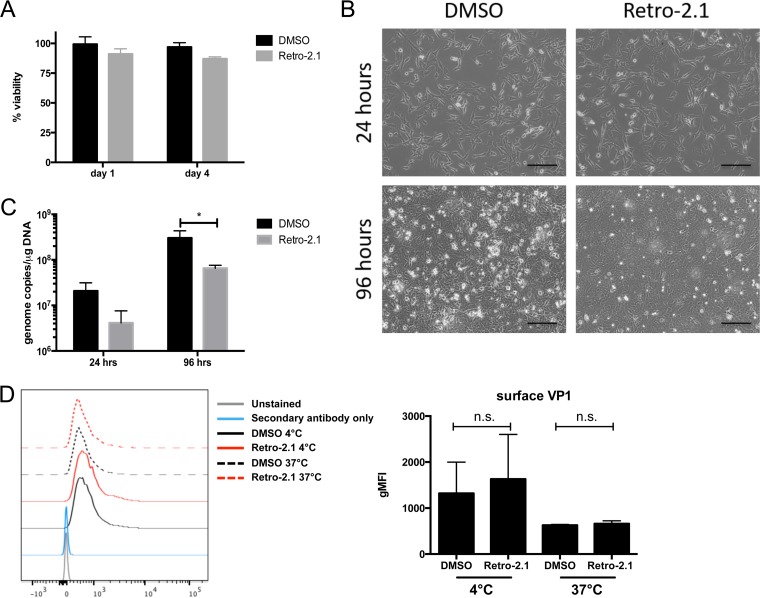
Retro-2.1 inhibits MuPyV infection without affecting host cell viability. (A) Viability of uninfected A31 cells in DMSO or 10 µM Retro-2.1 over 96 h using the PrestoBlue cell viability assay. (B and C) Subconfluent monolayers of A31 cells were infected at an MOI of 0.1 for 1.5 h at 37°C. The culture medium was supplemented with 10 µM Retro-2.1 or DMSO. (B) Bright-field images of infected cells treated with DMSO or Retro-2.1 at 24 and 96 h p.i. Bars = 200 µm. (C) Viral genome copies present in 10 ng of total cellular DNA. (D) (Right) Geometric mean fluorescence intensity (gMFI) of surface VP1 expression on A31 cells infected at an MOI of 3 for 1 h at 4°C or 30 min at 4°C followed by 30 min at 37°C. Values are means plus SD for three independent experiments. Values that are not significantly different (n.s.) are indicated.

Thus, 10 µM Retro-2.1 was used for all subsequent *in vitro* experiments. No loss of viability of uninfected A31 cells treated with Retro-2.1 was detected over a 96-h observation period (Fig. 2A). Subconfluent monolayers of A31 cells infected with MuPyV at a multiplicity of infection (MOI) of 0.1 in the presence of 10 µM Retro-2.1 attained confluence by 24 h and exhibited only modest virus-induced cytopathic effect (CPE) by 96 h p.i. compared to control cells treated with vehicle (Fig. 2B). This reduced CPE was associated with a significant decrease in the number of viral genome copies (Fig. 2C).

To determine whether Retro-2.1-mediated inhibition of MuPyV infection occurred at the level of virion binding to host cell receptors, A31 cells treated with Retro-2.1 or vehicle were exposed to MuPyV for 1 h at 4°C to prevent virion endocytosis. No change in cell surface staining by a monoclonal antibody (MAb) against the major viral capsid protein VP1 was noted between Retro-2.1- and vehicle-treated cells. When the cells were cultured at 37°C for 30 min after infection to allow internalization of bound virions, VP1 expression remained the same between vehicle- and Retro-2.1-treated cells (Fig. 2D). These data demonstrate that Retro-2.1 inhibits neither binding of virions to host cell receptors nor virion internalization, as was reported for the absence of Retro-2^cycl^ effects on JCPyV virion binding to host cells (18).

### Retro-2.1 treatment reduces viral burden in the kidneys during acute MuPyV infection.

**FIG 3:**
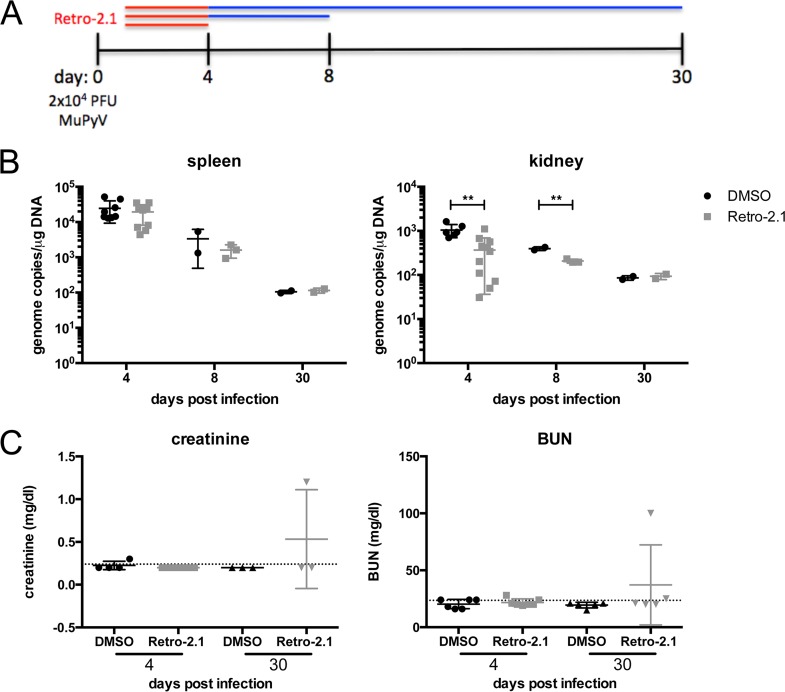
Kidney-specific decrease of viral burden in Retro-2.1-treated mice. (A to C) Mice were treated with 250 µg (12.5 mg/kg) Retro-2.1 or vehicle control 1 h prior to hind footpad infection with 2 × 10^4^ PFU MuPyV, followed by daily treatment until day 4 p.i. (B) Viral genome copies in the spleen and kidney at days 4 (*P* = 0.0012), 8 (*P* = 0.0051), and 30 p.i. (C) Serum creatinine and BUN from infected mice on day 4 and day 30 postinfection. The dotted lines indicate the baseline serum creatinine and BUN levels in uninfected adult C57BL/6 mice. The values are means ± SD for three to six independent experiments with two or three mice in each group.

Retro-2.1 is a highly lipophilic compound, necessitating solubilization in 100% dimethyl sulfoxide (DMSO). Optimization of dosing and delivery based on the solubility of the compound permitted a maximal single dose of 250 µg (12.5 mg/kg of body weight) administered intraperitoneally (i.p.). To examine whether Retro-2.1 inhibited MuPyV infection in mice, the following approach was used: healthy adult C57BL/6 mice were injected i.p. with 12.5 mg/kg of Retro-2.1 or DMSO 1 h before infection with 2 × 10^4^ PFU of MuPyV in their hind footpads, followed by daily injections of drug or vehicle control until day 4 p.i. (Fig. 3A). Mice were euthanized on day 4 p.i., and then their viral loads in kidneys and spleens were analyzed by qPCR. No difference in viral load was observed in the spleens of Retro-2.1- or vehicle-treated animals. However, a significant decrease in viral genome copies was seen in the kidneys of Retro-2.1-treated mice (Fig. 3B). A significant, albeit lower-magnitude, decrease in viral genome copies was present in the kidneys on day 8 p.i., when anti-MuPyV CD8 T cells infiltrate the kidneys and may contribute to controlling infection. These data demonstrate a kidney-specific inhibitory effect of Retro-2.1 on acute MuPyV infection.

To determine whether this Retro-2.1 treatment regimen altered kidney function, we assayed serum creatinine and blood urea nitrogen (BUN) in MuPyV-infected mice on days 4 and 30 p.i., according to the experimental design in Fig. 3A. No elevation in serum creatinine or BUN was observed in Retro-2.1-treated mice on day 4 p.i. (Fig. 3C), indicating that this regimen for administering Retro-2.1 did not impair renal function. At day 30 p.i., one drug-treated mouse did have significant renal toxicity, as evidenced by an increase of both creatinine and BUN; however, significant hemolysis in this sample may have contributed to the elevation in these markers or renal function (30). Thus, on the basis of serum creatinine and BUN assay results, Retro-2.1 did not cause a significant change in renal function.

### Anti-MuPyV CD8 T cell response is unaffected by Retro-2.1 treatment.

**FIG 4:**
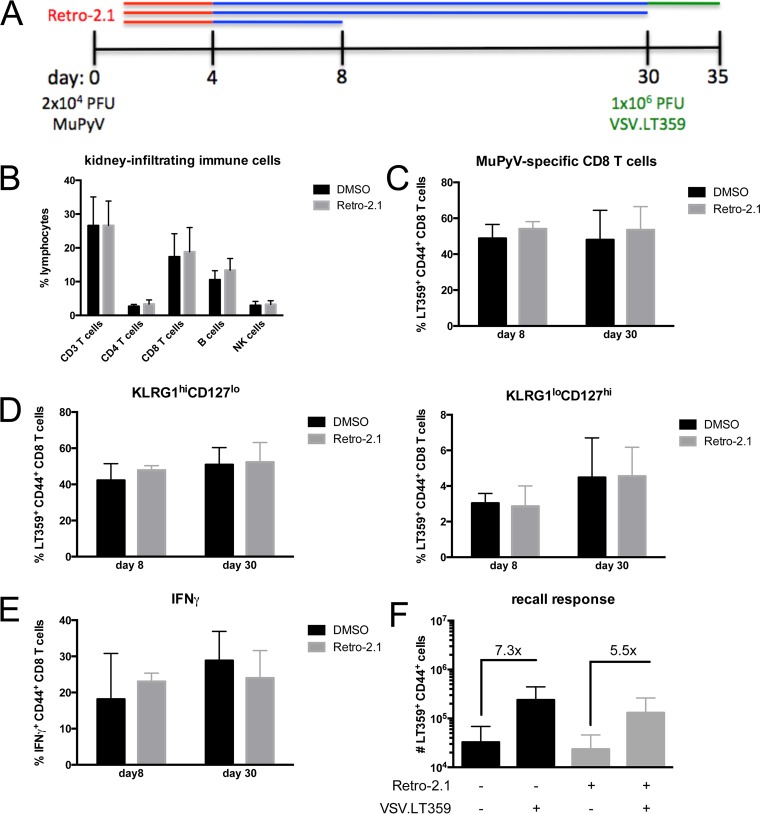
MuPyV-specific immune response is unaffected by Retro-2.1 treatment. (A) Mice were treated with 250 µg (12.5 mg/kg) Retro-2.1 or vehicle control 1 h prior to infection with 2 × 10^4^ PFU MuPyV, followed by daily treatment until day 4 p.i. The mice were euthanized at the indicated time points. (B) Percentage of total immune cells isolated from the kidneys on day 8 p.i. (C) Frequency of MuPyV-specific CD8 T cells in the kidney on day 8 and 30 p.i. (D) Percentage of MuPyV-specific CD8 T cells with KLRG1^high^ CD127^low^ (effector memory phenotype) and KLRG1^low^ CD127^high^ (central memory phenotype) phenotype. (E) IFN-γ expression in kidney CD8 T cells on day 8 and 30 p.i. stimulated for 5 h *ex vivo* with 1 µM LT359 peptide. (F) Mice were challenged with 1 × 10^6^ PFU of VSV.LT359 i.v. on day 30 p.i. and euthanized 5 days later. The number of MuPyV-specific cells in the kidneys with (+) and without (−) VSV.LT359 challenge infection is shown. Values are means plus SD for three independent experiments with three mice in each group.

We next evaluated whether Retro-2.1 affected the immune response to MuPyV infection. To this end, mice were infected and treated with 12.5 mg/kg of Retro-2.1 as depicted in Fig. 4A, and euthanized at the indicated time points. On day 8 p.i., no differences were seen in the accumulation of NK cells, B cells, or T cells in the kidneys of vehicle- and Retro-2.1-treated mice (Fig. 4B).

MuPyV-specific CD8 T cells are responsible for controlling infection (31, 32). The dominant MuPyV CD8 T cell response in C57BL/6 mice is directed against a peptide corresponding to amino acids 359 to 368 of the large T antigen presented by H-2D^b^; this T cell ligand is designated D^b^LT359. D^b^LT359-specific CD8 T cells were equivalent in frequency, KLRG1 and CD127 (interleukin 7 receptor alpha [IL-7Rα]) effector memory phenotype, and production of gamma interferon (IFN-γ) between Retro-2.1- and vehicle-treated mice in both acute (day 8 p.i.) and persistent (day 30 p.i.) phases of infection (Fig. 4C to E). We recently reported that the functionality of D^b^LT359-specific CD8 T cells is improved by reducing MuPyV load during acute and persistent infection (33). To determine whether treatment with Retro-2.1 augmented the antigen-specific recall response by D^b^LT359-specific CD8 T cells in persistently infected mice, we treated mice with Retro-2.1 or vehicle control daily until day 4 p.i. and infected the mice at day 30 p.i. with VSV.LT359, a recombinant vesicular stomatitis virus (VSV) expressing the LT359 epitope (34). As shown in Fig. 4F, both control and Retro-2.1-treated mice exhibited similar recall responses. Together, these data indicate that Retro-2.1 administered during acute infection does not impact the immune response to MuPyV infection.

### Retro-2.1 treatment reduces viral burdens in immunosuppressed mice.

**FIG 5:**
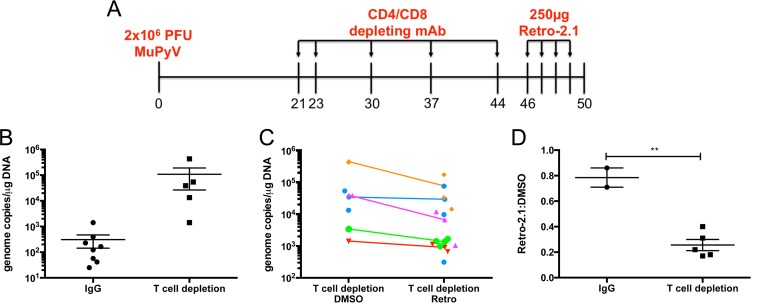
Retro-2.1 treatment limits increase in MuPyV in the kidneys of mice depleted of T cells during persistent infection. (A) Mice were infected with 2 × 10^6^ PFU MuPyV via the hind footpads at day 0, followed by administration of CD4 and CD8α MAbs from day 21 p.i. and continuing weekly to day 44 p.i. The mice were treated with 250 µg of Retro-2.1 (12.5 mg/kg) for 4 days and euthanized on day 50 p.i. (B) MuPyV genome copies in the kidneys of T cell-depleted and control mice on day 50 p.i. (C) Viral genome copies in the kidneys of Retro-2.1- or vehicle-treated mice with peripheral T cell depletion on day 50 p.i. Each symbol represents the value for one mouse, and each line represents the average reduction in genome copies in one independent experiment; each color corresponds with one experiment. (D) Fold change of viral genome copies from panel C. Values are means ± SD for three to five independent experiments with one to four mice in each group. The mean values are significantly different (*P* = 0.0041) as indicated by the bracket and two asterisks.

The natural disease progression of BKPyV-associated nephropathy (BKVAN) involves systemic immunosuppression and resurgence of persistent virus in kidney allografts. To induce this increase in MuPyV levels in persistently infected mice, CD4^+^ and CD8α^+^ cells were dually depleted with monoclonal antibodies starting at day 21 p.i., as depicted in Fig. 5A. T cell-depleted mice exhibited a 352-fold increase in viral genome copies in the kidneys compared to IgG-treated mice (Fig. 5B); notably, no increase in viral load was seen in the spleen or brain in these T cell-depleted mice (data not shown). This kidney-associated susceptibility to persistent MuPyV resurgence may stem from recent evidence that most kidney resident memory T cells are situated in the renal vasculature as marginated pools (35), a location rendering them more susceptible to systemic MAb-mediated depletion than in other organs. It is worth noting that the nature of PyV persistence is unknown; i.e., does the virus establish a dormant state akin to herpesvirus latency or maintain itself as a continuous low-level infectious state? Moreover, the nature and mechanisms by which depressed antiviral T cell responses set the stage for PyV “reactivation” remain to be determined.

Two days after the last injection of anti-CD4 and anti-CD8α, mice received either Retro-2.1 or vehicle control daily for 4 days, and virus genome copies were enumerated on the fifth day. Because of the sizeable range of viral load increases in the kidneys in T cell-depleted mice in separate experiments, viral genome copy numbers for vehicle- and Retro-2.1-treated groups were analyzed separately for each experiment; thus, each line shown in Fig. 5C represents one independent experiment. Mice that did not receive T cell depletion treatment showed no significant difference in kidney viral levels between DMSO vehicle- and Retro-2.1-treated mice. Notably, T cell-depleted mice given Retro-2.1 exhibited an approximately 4.2-fold reduction in virus levels compared to vehicle-treated control mice (Fig. 5D). Thus, in a clinically relevant scenario of PyV resurgence in a persistently infected host, an inhibitor of retrograde transport was capable of limiting viral infection.

## DISCUSSION

There is a pressing clinical need for anti-PyV therapeutics to control infection by PyVs in immunosuppressed individuals to prevent the onset or limit the progression of diseases caused by these viruses. Intracellular migration by AB_5_ toxins and PyV virions involves the retrograde vesicular transport pathway. Following identification of a dihydroxy quinazolinone inhibitor of retrograde transport that prevented Shiga toxin translation arrest *in vitro* and lethality in mice (16) and recognition that retrograde transport constituted an early step of the PyV lifestyle, Nelson and colleagues reported that the Retro-2^cycl^ compound and its derivatives inhibited BKPyV and JCPyV replication in tissue culture (18). Using the natural mouse pathogen, MuPyV, we demonstrate here that Retro-2.1, a Retro-2^cycl^ derivative with improved inhibition against Shiga toxin translation arrest (19), reduces MuPyV replication in kidneys without affecting kidney function or host immunity. Importantly, we further show that depletion of circulating T cells during persistent infection leads to resurgence of MuPyV in the kidneys, which is mitigated by administration of Retro-2.1. These data provide the first evidence for a compound having anti-PyV activity in a natural host.

Uncontrolled BKPyV replication in immunocompromised individuals is responsible for BKVAN, hemorrhagic cystitis (HC), and ureteral stenosis (US). BKVAN develops in approximately 10% of kidney transplant recipients who present with viremia and is characterized by lytic destruction of tubular epithelial cells, interstitial inflammation, tubular fibrosis and atrophy, functional impairment, and potential graft loss (2, 8). BKPyV-associated HC occurs in 24% of hematopoietic stem cell transplant (HSCT) recipients, presenting as hematuria, bladder pain, and dysuria more than 2 weeks after the transplant (36). US develops in approximately 6% of kidney transplant recipients (5). In all these patients, high viremia has been associated with an increased risk of disease progression, further emphasizing the need for an anti-PyV therapeutic.

Two clinical options are available when at-risk patients develop BKPyV viremia and/or BKPyV-associated diseases. The mainstay therapy is to reduce immunosuppression following the rationale that elevating adaptive immunity will control virus levels; for transplant patients with BKVAN, this approach increases the risk for allograft rejection. The second option is to administer antiviral drugs that have demonstrated anti-PyV activity in tissue culture. Cidofovir, a nucleoside analog, reduces BKPyV replication in primary human renal proximal tubule epithelial cells (37). However, meta-analysis has shown that cidofovir has no effect on viruria or viremia and does not favorably alter graft failure (38). A single-center retrospective study further suggested that >6 months of cidofovir administration was associated with a decline in graft function (39), likely as a result of its severe nephrotoxicity. Leflunomide, a pyrimidine synthesis inhibitor, was initially reported to reduce BKPyV levels in kidney transplant recipients (40). However, leflunomide, along with a reduction in immunosuppression, was determined by meta-analysis not to prevent graft failure (38), and a subsequent study showed no relationship between serum leflunomide concentration and viremia (14). Fluoroquinolones, broad-spectrum antibiotics that block type II and IV topoisomerases, also do not improve kidney allograft outcome and are instead associated with a higher risk of adverse events (41). The low efficacy and poor outcomes of these drugs may stem from their broad effects on processes essential for cellular viability.

Retro-2.1 is a specific inhibitor of retrograde transport, an early step in the PyV replication pathway (17, 42). Once endocytosed, vesicles containing single PyV virions transit to the endoplasmic reticulum (ER) where they are destabilized and then undergo retrotranslocation to the cytosol for partial uncoating. This process unveils nuclear localization signals that target the virion to the nucleus and allow viral replication to commence (17, 43, 44). Nelson and colleagues have shown that Retro-2^cycl^ prevents localization of BKPyV virions to the endoplasmic reticulum, thereby inhibiting spread of infection *in vitro* (18).

A hurdle to administering any of the Retro family derivatives formulated thus far is their pronounced hydrophobicity (28, 29). These drugs are typically solubilized in 100% DMSO and delivered *in vivo* in a 0.9% saline formulation; this poor aqueous solubility limits the amount of drug that can be delivered. Accounting for the maximal dose (250 µg) in the maximal volume (1 ml) that can be delivered i.p., we were able to deliver 12.5 mg/kg of drug daily. With this dosage, we found that Retro-2.1 significantly reduced kidney virus levels in MuPyV-infected mice. The lack of antiviral activity in the spleen may stem from the high levels of viral replication during acute infection in this organ following hind footpad inoculation. This possibility is supported by data that the antiviral effect of this dose or Retro-2.1 in mice inoculated with 2 × 10^4^ PFU of MuPyV was not seen with an inoculation dose of 2 × 10^6^ PFU (data not shown). During persistent MuPyV infection, viral DNA levels in the kidney are close to the limit of detection, rendering potential antiviral effects difficult to detect.

Detection of Retro-2.1’s anti-MuPyV activity only in the kidney may additionally be due to concentration of the compound by the renal plasma filtration apparatus, which would expose the uroepithelium to higher doses of Retro-2.1 than can be achieved systemically. Following from this possibility, it is worth emphasizing that pharmacokinetic studies for Retro-2.1 remain to be performed to define optimal dosing regimens that fit within an anti-PyV therapeutic window while minimizing potential toxicity. Poor aqueous solubility clearly obviates use of this Retro-2.1 as an antiviral therapeutic for human PyVs. Evidence presented here should motivate efforts to develop derivatives of this compound with improved aqueous solubility or to encapsidate the derivates in nanoparticles to permit intravenous (i.v.) infusion and possibly extend the tissue range for their antiviral activity.

In summary, we have used the MuPyV mouse infection model to demonstrate the *in vivo* efficacy of Retro-2.1 as an anti-PyV agent. Retro-2.1 has low cellular toxicity and high specificity for inhibiting retrograde vesicular intracellular trafficking, which suggests that a therapeutic formulation of the drug would carry a low risk of adverse effects. Notably, Retro-2^cycl^ and Retro-2.1 have also been shown to act on a wide variety of pathogens in addition to PyVs, including Leishmania major, human papillomavirus, human and mouse cytomegaloviruses, enterovirus, and Ebola and Marburg viruses (45–49). The expanding use of immunomodulatory therapies to control inflammatory and autoimmune disorders, administration of immunosuppressive agents with increased potency, and the increasing size of the transplant patient population raise the specter of an increasing incidence for life-threatening PyV-associated diseases. The development of potent and effective anti-PyV agents, of which Retro compounds are a promising candidate, is a high biomedical priority.

## MATERIALS AND METHODS

### Mice and *in vivo* treatment.

Female C57BL/6 were purchased from the National Cancer Institute (Frederick, MD) and used at 7 to 14 weeks of age. The mice were injected intraperitoneally (i.p.) with 250 µg (12.5 mg/kg of body weight) of Retro-2.1 in 1 ml of 0.9% saline 1 h prior to infection. The A2 laboratory strain of MuPyV (2 × 10^4^ PFU) was injected in 100-µl volume into the hind footpads. The mice were given 12.5 mg/kg Retro-2.1 once a day as specified. All mice were housed in the Penn State College of Medicine vivaria and used in accordance with an approved Penn State Hershey Institutional Animal Care and Use Committee protocol.

### Retro derivatives.

Retro-2^cycl^ and Retro-2.1 were synthesized by the Organic Synthesis Core at the Penn State College of Medicine. The products were purified by silica gel column chromatography, and purity was determined by analytical high-performance liquid chromatography (HPLC). The structures of intermediates and final compounds were characterized by nuclear magnetic resonance (NMR) and mass spectra. The analytical characteristics of the compounds were identical to those reported earlier (19). The level of purity of ≥98% for the final compounds was achieved after chromatography or chromatography followed by crystallization.

### Cells and infection.

BALB/3T3 clone A31 cells (American Type Culture Collection, Manassas, VA) were cultured in Dulbecco modified Eagle medium (DMEM) supplemented with 10% fetal bovine serum (FBS), 100 U/ml penicillin, and 100 µg/ml streptomycin. Subconfluent monolayers were infected at a multiplicity of infection (MOI) of 1 or 0.1 for 1.5 h at 37°C. Following infection, virus was aspirated and cells were replenished with 10% DMEM containing the indicated concentration of Retro-2^cycl^ or Retro-2.1.

### Viability assay.

Cell viability was evaluated using the PrestoBlue cell viability reagent (Invitrogen, Carlsbad, CA). Briefly, cells were incubated in 96-well culture plates for 96 h in the presence of drug in a total volume of 90 µl. At the indicated time points, cells were incubated with 10-µl reagent for 10 min at 37°C, and then the wells were immediately read on a spectrophotometer at 560 nm.

### Virus and synthetic peptides.

The A2 laboratory strain of MuPyV was prepared in baby mouse kidney cells as described previously (50). A recombinant vesicular stomatitis virus carrying the LT359-638 sequence (VSV.LT359) was generated as described previously (34). The titers of the virus were determined by a plaque assay on A31 cells as previously described (51). Mice were infected in hind footpads with 100 µl of virus. An HPLC-purified peptide corresponding to residues 359 to 368 of the large T antigen (SAVKNYCSKL) was synthesized by Peptide 2.0, Inc. (Chantilly, VA), dissolved in phosphate-buffered saline (PBS), and stored at −20°C.

### Viral genome quantitation.

TaqMan real-time PCR was performed using 10-ng template DNA purified from snap-frozen tissues using the Maxwell 16 nucleic acid isolation system (Promega, Madison, WI) as previously described (52).

### Viral large T antigen mRNA quantitation.

Total RNA was isolated from cultured cells using TRI reagent (Sigma-Aldrich, St. Louis, MO) per the manufacturer’s instructions. Two micrograms of RNA was converted to cDNA using RevertAid H minus reverse transcriptase (Thermo Scientific, Waltham, MA) per the manufacturer’s instructions. Viral large T antigen (LT-Ag) mRNA was quantified by quantitative PCR (qPCR) with FastStart Universal Probe Master (ROX) mix (Roche) using an ABI PRISM 5700 sequence detection system (Applied Biosystems, Foster, CA) using a forward primer (5′-AGGAATTGAACAGTCTCTGGG-3′), reverse primer (5′-GTCATCGTGTAGTGGACTGTG-3′), and TaqMan probe (5′-AGAGCCCTGGAAGCCGGTT-3′) (Integrated DNA Technologies, Coralville, IA). Validated TATA box-binding protein (*Tbp*) gene TaqMan primers from Integrated DNA Technologies were used as a housekeeping control gene for normalization of LT-Ag mRNA threshold cycle (*C*_*T*_) values. A standard curve of known copy number for LT-Ag mRNA was performed using a plasmid containing a cloned LT-Ag mRNA amplicon.

### T cell isolation and flow cytometry.

The spleens and kidneys were harvested on the indicated days postinfection (p.i.). The kidneys were minced and digested with collagenase (100 U/ml in DMEM with 2% FBS, 200 U/ml penicillin, 200 µg/ml streptomycin, 2 mM l-glutamine, 5 µM HEPES, 1 µM MgCl_2_, 1 µM CaCl_2_) for 30 min at 37°C, passed through a 70-µm nylon cell strainer (BD Biosciences, San Jose, CA). The cells were isolated by centrifugation on a 44% and 66% Percoll gradient. The spleens were passed through a 70-µm nylon cell strainer and then treated with ACK buffer (0.15 M NH_4_Cl, 1 mM KHCO_3_, 1 mM Na_2_EDTA [pH 7.0]) to lyse red blood cells (RBCs). The cells were exposed to fixable viability dye eFluor 780 (eBioscience, San Diego, CA) and then surface stained in fluorescence-activated cell sorting (FACS) buffer (PBS [pH 7.2] with 1% bovine serum albumin [BSA] and 0.1% sodium azide) for 30 min at 4°C with H-2D^b^ LT359 tetramers (provided by the NIH Tetramer Core Facility, Atlanta, GA) and monoclonal antibodies (MAbs) to CD19, NK1.1, CD3, CD8α (clone 53-6.7; BioLegend, San Diego, CA), CD44 (clone IM7; eBioscience), KLRG1 (clone 2F1; BD Biosciences), and CD127 (clone AFR34; BioLegend). The samples were collected on a BD LSRFortessa or BD FACSCanto10 flow cytometer (San Jose, CA). Fluorescence-minus-one (FMO) samples were used to set positive gates for each surface molecule examined.

### Intracellular MAb staining.

Splenocytes were cultured for 5 h in DMEM containing 10% FBS, 100 U/ml penicillin, and 100 µg/ml streptomycin and supplemented with 1 µg/ml brefeldin A (Sigma-Aldrich). The cells were exposed to fixable viability dye eFluor 780 (eBioscience), surface stained with MAbs to CD8α (clone 53-6.7; eBioscience) and CD44 (clone IM7; eBioscience), permeabilized with Cytofix/Cytoperm buffer (BD Biosciences), and stained for intracellular gamma interferon (IFN-γ) (clone XMG1.2; BioLegend).

### Blood assays for kidney function.

Two hundred microliters of whole blood was obtained from the submandibular vein via cheek bleed and immediately transported to the clinical laboratory of the Department of Comparative Medicine of the Penn State College of Medicine for analysis. Serum was used to analyze blood urea nitrogen (BUN) and creatinine using the Cobas Mira Plus chemistry analyzer (Roche Diagnostics Systems, Indianapolis, IN).

### T cell depletion.

Mice were injected i.p. with 250-µg rat anti-CD8α (YTS169.4; Bio X Cell, West Lebanon, NH), rat anti-CD4 (GK1.5; Bio X Cell, West Lebanon, NH), or ChromPure whole-rat IgG (Jackson ImmunoResearch Laboratories, West Grove, PA) at 21, 23, 30, 37, and 44 days p.i. T cell depletion was confirmed in blood by flow cytometry using Absolute Count Standards (Bangs Laboratories, Fishers, IN).

### Memory CD8 T cell recall.

Persistently infected mice (day 30 p.i.) were infected i.v. with VSV.LT359 and sacrificed 5 days postchallenge to evaluate the recall response of LT359-specific CD8 T cells.

### Statistical analysis.

All data are displayed as means ± standard deviations (SD). *P* values were determined using an unpaired Student’s *t* test assuming equal variance or one-way analysis of variance (ANOVA) using GraphPad Prism software (La Jolla, CA). *P* values of ≤0.05 were considered significant.
